# Trends of stillbirths in Harare City, Zimbabwe, 2015-2019: a secondary data analysis

**DOI:** 10.11604/pamj.2022.43.117.34677

**Published:** 2022-11-01

**Authors:** Talent Bvochora, Hilda Bara, Mujinga Karakadzai, Addmore Chadambuka, Tsitsi Juru, Prosper Chonzi, Notion Gombe, Mufuta Tshimanga

**Affiliations:** 1 University of Zimbabwe, Department of Primary Health Care Sciences, Global and Public Health Unit, Harare, Zimbabwe,; 2 Harare City Health Department, Harare, Zimbabwe,; 3 African Field Epidemiology Network, Harare, Zimbabwe

**Keywords:** Stillbirth, trends, Zimbabwe

## Abstract

**Introduction:**

in Zimbabwe, perinatal mortality is a major public health problem. Harare City data showed increase in stillbirth rate trend from 4/1000 live births in 2014 to 6/1000 live births in 2018, failing to meet the country’s target of reducing stillbirth rate by 40%. We analysed the characteristics of stillbirths from 2015 to 2019 in Harare City.

**Methods:**

we conducted a retrospective analytical cross-sectional study using secondary data from Harare City Health Department’s 12 baby-delivery polyclinics. Fourteen key informants were interviewed to verify information obtained. Using Epi-info, descriptive summaries and graphs were generated and bivariate and multivariate logistic regression was conducted. Statistical significance was considered at a p-value <0.05.

**Results:**

a total of 700(74.9%) perinatal death notification records were reviewed. The majority were macerated stillbirths 418(59.7%) followed by fresh stillbirths 189(27.0%). The median age for women who had fresh stillbirths was 26 years (Q_1_=22; Q_2_=32). Preterm delivery (aOR= 2.15; 95%CI 1.81- 3.89; p<0.01), having delivered by breech presentation (aOR= 3.32; 95%CI 1.72-6.41; p=<0.01), and being HIV positive (aOR= 1.69; 95%CI 1.02-2.79; p=0.04) were associated with preterm delivery.

**Conclusion:**

stillbirths in Harare City were increasing and were due to preventable causes. The younger maternal age group was most affected hence preventive activities should focus on them. Improving the quality of antenatal care, delivery, and new-born care can help reduce stillbirths and early neonatal death.

## Introduction

In low-income countries such as Zimbabwe, perinatal mortality remains a major public health problem with most causes being preventable [1]. The World Health Organization (WHO) defines perinatal mortality as deaths occurring during late pregnancy (>22 weeks of gestation), during birth, and within seven days after delivery with a stillbirth specifically defined as delivery of a baby with no signs of life from 28 weeks of gestation[2]. The perinatal period reflects the general health and the various socio-biological features of mothers and babies [3]. Global estimates show that 3.3 million stillbirths occur every year with one in three of these deaths occurring during delivery [3]. Stillbirths lead to long-standing parental depression and are also distressing to the healthcare workers. A population-based case-control study showed that women who had a stillbirth were nearly twice as likely to report depressive symptoms months after delivery than women who had a live birth [4]. Intrapartum stillbirths are largely preventable with quality care which includes prompt recognition and management of labour complications [5].

In the less developed countries, which account for 98% of perinatal deaths, these deaths are not always registered [6]. Third-trimester stillbirth rates in sub-Saharan Africa are approximately ten times that in developing countries [7]. Sustainable Development Goal (SDG) 3 aims to end preventable new-born mortality and stillbirths by 2030 [8]. The perinatal mortality rate for Zimbabwe was estimated to be around 34 per 1000 births according to the Zimbabwe Demographic Health Survey of 2015 [9]. Studies have shown that in Zimbabwe, women seek antenatal care late and prioritise first-child pregnancies compared to the subsequent pregnancies [10,11]. Reasons for late booking include lack of information of the purpose of early antenatal care, long waiting time and unfriendly services. The country aims to achieve global targets of a stillbirth rate of 12 per 1000 births or less by 2030.

In Zimbabwe, the implementation of the perinatal death notification system started in 1986. Initially, it was a paper-based system, however, it is now electronic from district level upwards. The perinatal death reporting form is completed for all perinatal deaths that occur, at the facility were the death occurred. These forms also include stillbirth records. In Harare City health department, stillbirths are captured using the perinatal notification forms at the twelve polyclinics that offer maternity services. The form is completed in triplicate within 7 days of perinatal death. Variables that are captured include age, marital status, religion, parity, gravida, booking status, and cause of death. A copy is kept at the health facility and the other 2 copies are submitted to the provincial health information office through the district health information officer, from where a copy is submitted to the national reproductive health unit. This procedure is adapted from the national level reporting system.

**Figure 1:**
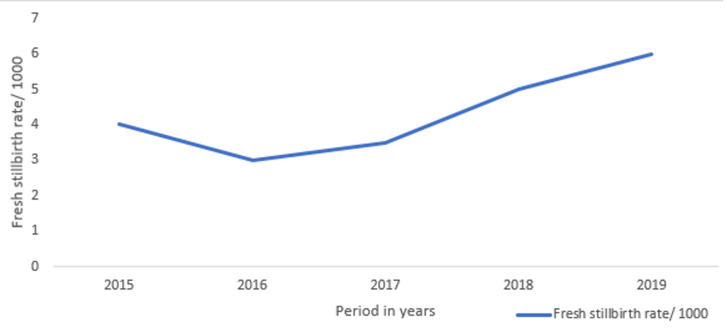
fresh stillbirth rate/1000

Harare City health department data showed an increase in stillbirth (SB) rate trends from 4/1000 live births in 2014 to 6/1000 live births in 2018 as shown in Figure 1. Despite various interventions such as training in Emergency Obstetric and Neonatal care (EMNOC), capacitation of frontline health care workers through midwifery manpower development, offering family planning, adopting the focused antenatal care model, and procurement of equipment and sundries, Harare City health department has failed to reduce the negative outcomes and has also failed to meet the country´s target of reducing the SB rate by 40%. It was therefore crucial to analyse the characteristics of stillbirths from 2015 to 2019 in Harare City. This would help to identify the preventable drivers and come up with measures to reduce the SB rate.

## Methods

**Study design:** a retrospective analytical cross-sectional study using secondary data was conducted.

**Study setting:** Harare Metropolitan Province of Zimbabwe has a population of 1,973,906 according to ZIMSTAT 2017. Harare City Health Department is run under Harare Municipal authority as a parastatal to the government of Zimbabwe. The department has two infectious diseases hospitals (Wilkins and Beatrice Road), an emergency services center, and 43 clinics which are distributed in its four districts. There are 12 polyclinics in Harare City which offer baby delivery services. The study was carried out using data collected from these 12 polyclinics.

**Source of data:** an electronic perinatal mortality database and delivery registers from 2015-2019 were retrieved and used in the analysis. Fourteen key informants, including the sisters in charge, a matron and district medical officers were interviewed to verify the information obtained from the database and registers.

**Variables:** the dependent variable in this study was stillbirth (outcome). The independent variables (exposures) were broken down into categories namely booking status (not booked), maternal medical conditions (hypertension, HIV) and types of delivery (breech delivery).

**Data analysis:** an electronic questionnaire was created and data were captured and analyzed using Epi Info 7.2.4.0™ (CDC, 2020) statistical software. Data were entered, cleaned for transcription errors, missing details, duplicate information, and values that were out of range. Descriptive summaries and graphs were generated for trends of stillbirths from January 2015 to December 2019. Bivariate analysis was used to determine the strength of association between the independent variables (booking status, maternal conditions and types of delivery) and the dependent variable (stillbirth). Crude odd ratios (OR) and 95% confidence intervals were calculated. Statistical significance was considered at a p-value <0.05 and only significant variables were entered into multivariate logistic regression to identify factors independently associated with stillbirth.

**Permission and ethical considerations:** permission to carry out the study was obtained from the Harare City Institutional Review Board and Health Studies Office. To maintain confidentiality, the names of the mothers and babies were not used. Data is kept under lock and key.

## Results

A total of 700(74.9%) of the perinatal death notification records were reviewed from the delivery registers. Of these, the majority were macerated stillbirths (MSBs) 418(59.7%) followed by fresh stillbirths (FSBs) 189(27.0%). The median age for the women was generally similar with women who had FSBs having a median age of 26 years (Q_1_=22; Q_2_=32). The socio-demographic characteristics of women who delivered stillbirths or had early neonatal deaths (ENND) at maternity departments of Harare City, 2015- 2019 are summarised in Table 1. Most of the mothers lived in the high-density areas where all the polyclinics are situated. The majority of the mothers were in their second and third pregnancies with most of them having two or fewer children. Of the 418 mothers who had MSBs, 277(66.3%) were booked whilst for 189 mothers who had FSBs, 152(80.4%) were booked. Most of the stillbirths were males. Two hundred and thirty-one (55.3%) of MSBs were males. One hundred and fourteen (60.3%) of FSBs were female. Most of the MSBs 279(66.8%) were less than 2500 grams in weight whilst most of the FSBs 121(64.0%) were more than 2500 grams.

**Table 1:** socio-demographic characteristics of women who delivered stillbirths or had early neonatal deaths at maternity departments of Harare City Council, Zimbabwe, 2015-2019

Variable	Category	Macerated n= (%)	Fresh n= (%)	ENND n= (%)
**Age**	< 20 years	34 (8.1)	24 (12.7)	12 (12.9)
	20-24 years	111 (26.6)	50 (26.5)	28 (30.1)
	25-34 years	195 (46.7)	87 (46.0)	42 (45.2)
	≥35 years	78 (18.7)	28 (14.8)	11 (11.8)
**Total**		418 (59.7)	189 (27.0)	93 (13.3)
**Median age= n (Q_1_, Q_3_)**		28 (22, 33)	26 (22, 32)	26 (22, 31)
**Residence**	High density	375 (89.7)	160 (84.7)	77 (82.8)
	Medium density	26 (6.2)	16 (8.5)	12 (12.9)
	Low density	17 (4.1)	13 (6.9)	4 (4.3)
**Gravida**	1	98 (23.4)	52 (27.5)	26 (28.0)
	2-3	213 (51.0)	102 (54.0)	44 (47.3)
	≥4	107 (25.6)	35 (18.5)	23 (24.7)
**Parity**	0	106 (25.4)	56 (29.6)	30 (32.3)
	1-2	223 (53.3)	104 (55.0)	45 (48.4)
	≥3	89 (21.3)	29 (15.3)	18 (19.4)
**Booking status of the mother**	Booked	277 (66.3)	152 (80.4)	67 (72.0)
	Unbooked	141 (33.7)	37 (19.6)	26 (28.0)
**Sex of foetus**	Male	231 (55.3)	114 (60.3)	51 (54.8)
	Female	187 (44.7)	75 (39.7	42 (45.2)
**Birth weight**	<2500 grams	279 (66.8)	68 (36.0)	39 (41.9)
	≥2500 grams	139 (33.3)	121 (64.0)	54 (58.1)

**Key informant interviews:** a total of fourteen key informants were interviewed. Key informants reported that the health department was supported by two ambulances. The data was captured on paper and then transferred to the electronic system at the provincial level. Health care workers were trained on obstetric emergencies twice a year. Attrition of staff was high.

**Figure 2:**
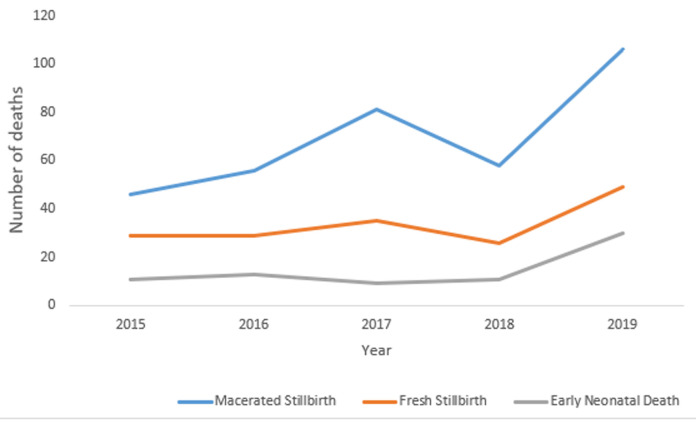
trends in macerated stillbirths, fresh stillbirths, and early neonatal deaths in Harare City 2015- 2019

**Trends in macerated stillbirths, fresh stillbirths, and early neonatal deaths in Harare City 2015-2019:** there was a steady increase in both FSBs and ENNDs from 2015 to 2016 in Harare City as shown in Figure 2. This was not statistically significant (p-value = 0.50). The MSB trend showed a rise from 2015 to 2017 then a sharp decline to 2018 and a sharp rise to 2019. This was not statistically significant (p-value = 0.504).

**Figure 3:**
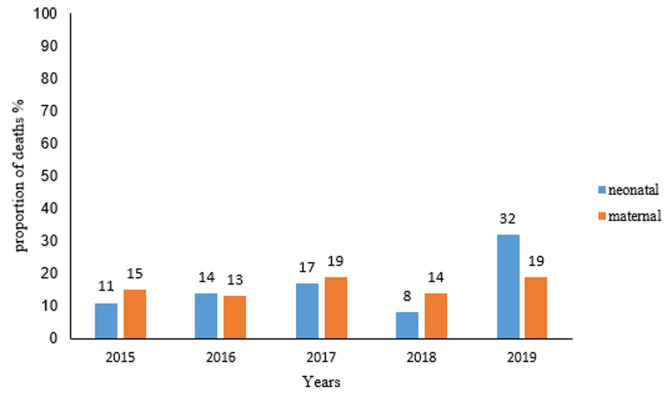
trends in stillbirths by neonatal and maternal causes

**Causes of stillbirths in Harare City, 2015- 2019:** most of the causes of stillbirths were not documented 460(65.7%) or were unknown 98(14%). The documented causes were fetal causes 145 (20.7%) and maternal causes 95(13.6%). The commonest maternal condition in those who had MSBs was a hypertensive disease with 80.4% (n=55). Cord prolapse was the most common maternal condition for FSBs at 15/29. Fetal distress was the commonest fetal cause of FSBs. Causes of stillbirths in Harare City Health Department are summarized in Table 2. In 2019, which recorded most stillbirths, fetal causes contributed more than maternal causes as illustrated in Figure 3.

**Table 2:** causes of stillbirths in Harare City, Zimbabwe, 2015-2019

Variable	Category	Macerated n= (%)	Fresh n= (%)	ENND n= (%)
Maternal causes	Hypertensive disorders	45 (80.4)	12 (41.4)	3 (30.0)
	Bleeding	5 (8.9)	2 (6.9)	3 (30.0)
	Maternal infections	4 (7.1)	0 (0)	1 (10.0)
	Cord prolapse	1 (1.8)	15 (51.7)	3 (30.0)
Fetal causes	Fetal distress		30 (46.9)	19 (42.2)
	Preterm: extremely (<28 weeks)	17 (4.1)	5 (2.7)	6 (6.5)
	(28- 32 weeks)	70 (16.8)	24 (12.7)	8 (8.6)
	moderate to late (32-37 weeks)	175 (41.9)	39 (20.6)	22 (23.7)
	Birth asphyxia		3 (4.7)	7 (15.5)
	Undiagnosed twin	20 (55.5)	14 (21.8)	2 (4.4)
	Fetal abnormalities	2 (5.6)	2 (3.1)	2 (5.6)

**Association between booking status and fresh stillbirth and early neonatal deaths at Harare City maternity departments, 2015- 2019:** mothers who had booked were less likely to deliver a FSB than those who had not booked, prevalence odds ratio (OR)= 0.57; confidence interval (CI)= 0.40- 0.80 and p= 0.001. Table 3 summarizes the association between booking status and FSB and ENND at Harare City maternity departments, 2015-2019.

**Maternal factors associated with stillbirths at Harare City maternity departments, 2015-2019:** mothers who were hypertensive were more likely to deliver an MSB than a FSB with an OR= 0.15; CI= 0.1- 0.38; p<0.01. Being HIV negative was protective against delivering a MSB OR= 0.56; CI 0.35- 0.88; p= 0.01. Preterm delivery was associated more with MSBs than FSBs, OR= 0.35; CI= 1.34- 1.75; p<0.01. Delivering normally was protective from FSBs than breech delivery, OR=0.32; CI 0.17- 0.60; p<0.01. Maternal factors associated with stillbirths in Harare City maternity departments, 2015-2019 are summarized in Table 3.

**Table 3:** factors associated with fresh stillbirth and early neonatal deaths at Harare City Maternity Department, 2019

Factor	Variable	Category	FSB and NND	MSB	OR	95% CI	P-value
**Booking status**	Booked		219	277	0.57	0.40-0.80	0.001
	Not booked		63	141			
**Maternal factors**	Presence of hypertension	Yes	15	45	0.15	0.1-0.38	<0.01
		No	24	11			
	HIV status	Positive	32	67	0.56	0.35-0.88	0.01
		Negative	208	243			
	Preterm delivery	Yes	104	262	0.35	0.25-0.48	<0.01
		No	178	156			
	Type of delivery	NVD*	251	402	0.32	0.17-0.60	<0.01
		Breech delivery	31	16			

* NVD: normal vaginal delivery; FSB: fresh stillbirths; ENND: early neonatal deaths; MSB: macerated stillbirths; CI: confidence interval; aOR adjusted odds ratio

**Independent factors associated with FSB and ENND in Harare City Health Department, 2015-2019:** independent factors found to be significantly associated with FSBs and ENND were not having had a preterm delivery (aOR= 2.15; 95%CI 1.81- 3.89; p<0.01), having delivered by breech presentation (aOR= 3.32; 95%CI 1.72-6.41; p=<0.01), and being HIV positive (aOR= 1.69; 95%CI 1.02-2.79; p=0.04) as shown in Table 4.

**Table 4:** independent factors associated with fresh stillbirths, and early neonatal deaths in Harare City Health Department, 2015-2019

Variable	aOR	95 % CI	P-value
Preterm delivery	2.15	1.81-3.49	<0.01
Breech delivery	3.32	1.72-6.41	<0.01
HIV positive	1.69	1.02-2.79	0.04

CI: confidence interval; aOR adjusted odds ratio

## Discussion

Our study is an assessment of the trends in SB rates over a five-year period in Harare City maternity clinics. The study also determined the maternal and neonatal factors associated with stillbirths in the capital city of Zimbabwe, Harare. Our study findings were that a steady increase in stillbirths in Harare City from 2015 and 2019 occurred mostly among women less than 34 years, with 3 or fewer children, and residing in high-density areas. Foetal distress was the most common cause of stillbirths. An association was found between preterm delivery, breech delivery and stillbirths as well as maternal conditions and stillbirths. Our study showed a steady rise in stillbirths from 2015 to 2019. With the maternal age group most affected being between 25 and 34 years, this could be explained by that this age group was seeking medical treatment late thinking they are now experienced in child bearing. This is also supported by that most mothers who had stillbirths were in their second or third pregnancy. The trend we observed was different from that observed from 2010- 2016 in a study done in developing countries, were there was a decline in mean SB rates, but the decline was low [12]. In Ghana, a study reported an overall decline in SB rate from 4.2% in 2003 to 2.1% in 2013, unlike in our study, but the years were different [13]. Similar to our study, a more recent study on stillbirth trends from 2000-2019 found no decrease in SB rates in sub-Saharan Africa [14].

Maternal age at birth has been reported as a contributor to stillbirth in developing countries [15,16]. Our finding that mothers aged less than 34 years experienced stillbirths could be due to a lack of knowledge on the danger signs of pregnancy and younger mothers having less experience in childbearing than the older women. This is unlike in other studies that were done in Uganda and Australia, where there was a higher probability of having a stillbirth in advanced age above 35 years [10,17]. In our study the risk of stillbirths was lower among teenage mothers compared to mothers from 20 to 34 years. This could be explained by the availability of programmes that target adolescent mothers in the communities or that teenage mothers were not reporting to health facilities at all in fear of legal issues around pregnancy. There was however a higher risk of stillbirths in teenage mothers in studies done in Uganda and previously in Zimbabwe [10,18].

In our study, most of the causes for stillbirths were not documented and some were documented as unknown. This may have been because health professionals were either too overwhelmed with work to document, or not confident in what could have caused the stillbirth. Key informants reported that training was being carried out but might not have adequate information to equip the health workers on documentation of causes of stillbirths. Studies have shown that there is poor knowledge on the causes and prevention of stillbirths among health care workers [19]. Lack of training in managing stillbirths was reported to leave health professionals ill-equipped to support mothers and provide responsive care [20]. Knowledge and attitudes towards key essential practices, such as the use of a partograph (labour monitoring tool) to assess labour progress, early initiation of breastfeeding, skin-to-skin care, and weighing the baby, improved among skilled birth attendants in Uganda [21].

The common causes attributed to FSBs and ENNDs in our study were preventable ones, namely birth asphyxia, foetal distress, and cord prolapse. This may have been due to sub-optimal monitoring during labour by the health care workers and also a delay in transfer to a higher level of care. Training in emergency management of obstetric complications is done at least yearly in Harare City health department. It was noted by key informants that the 12 facilities in the city were serviced by only two ambulances hence delays in transfer. Where health facilities exist and are accessible, the quality of health care offered may be poor due to understaffing, de-motivated health personnel, shortage of neonatal resuscitation equipment, or delayed referral systems due to transport challenges and poor communication network [22,23]. These could also be contributing factors in our study.

Like other studies, our study showed an association between prematurity and stillbirths. This could have been due to lack of or poor monitoring during antenatal period hence missing preventable causes. Antenatal care can potentially serve as a platform to deliver interventions to improve maternal nutrition, promote behaviour change to reduce harmful exposures and risk of infections, screen for and treat risk factors, and encourage skilled attendance at birth [24]. Breech delivery was also associated with FSBs. The unavailability of ultrasound scan services at the delivery facilities contributed to undiagnosed breech presentation and undiagnosed twin pregnancies which are normally referred to a higher institution for delivery. The need for quality antenatal care involving monitoring mothers and scans to identify maternal conditions and manage them appropriately and timeously would help reduce stillbirths. Studies have shown that breech deliveries were 4.7 times more likely to be a stillbirth compared to normal deliveries and operative delivery, especially caesarean section, contributes to decreased fresh stillbirth rates in breech presentation [18,25].

**Limitations:** the electronic records did not capture the cause of death, maternal condition of the mother, birth weight and gestational age. We therefore had to extract the information from the individual delivery registers at the facilities. Some of the registers were torn and some missing hence 25% of the records were not reviewed.

## Conclusion

Stillbirths in Harare City were increasing from 2015 to 2019 and were mainly due to preventable causes. The younger maternal age group was most affected and hence preventive activities should focus on this age group. Investment in delivery facilities to improve the quality of antenatal care, delivery, and new-born care can help reduce stillbirths and early neonatal deaths.

### What is known about this topic

- The major causes of stillbirth, including childbirth complications, post-term pregnancy, maternal infections during pregnancy (malaria, syphilis, and HIV), maternal disorders (especially hypertension, obesity, and diabetes), foetal growth restriction, and congenital abnormalities are known.

### What this study adds

- The younger maternal age group was most affected by stillbirths;
- The study adds supportive evidence that booking status and maternal conditions are associated with stillbirths.
